# Biodegradable Nanoparticle-Entrapped Vaccine Induces Cross-Protective Immune Response against a Virulent Heterologous Respiratory Viral Infection in Pigs

**DOI:** 10.1371/journal.pone.0051794

**Published:** 2012-12-11

**Authors:** Varun Dwivedi, Cordelia Manickam, Basavaraj Binjawadagi, Dechamma Joyappa, Gourapura J. Renukaradhya

**Affiliations:** 1 Food Animal Health Research Program, Ohio Agricultural Research and Development Center, The Ohio State University, Wooster, Ohio, United States, and Department of Veterinary Preventive Medicine, The Ohio State University, Columbus, Ohio, United States of America; 2 Foot and Mouth Disease Laboratory, Indian Veterinary Research Institute, Hebbal, Bengaluru, India; Virginia Polytechnic Institute and State University, United States of America

## Abstract

Biodegradable nanoparticle-based vaccine development research is unexplored in large animals and humans. In this study, we illustrated the efficacy of nanoparticle-entrapped UV-killed virus vaccine against an economically important respiratory viral disease of pigs called porcine reproductive and respiratory syndrome virus (PRRSV). We entrapped PLGA [poly (lactide-co-glycolides)] nanoparticles with killed PRRSV antigens (Nano-KAg) and detected its phagocytosis by pig alveolar macrophages. Single doses of Nano-KAg vaccine administered intranasally to pigs upregulated innate and PRRSV specific adaptive responses. In a virulent heterologous PRRSV challenge study, Nano-KAg vaccine significantly reduced the lung pathology and viremia, and the viral load in the lungs. Immunologically, enhanced innate and adaptive immune cell population and associated cytokines with decreased secretion of immunosuppressive mediators were observed at both mucosal sites and blood. In summary, we demonstrated the benefits of intranasal delivery of nanoparticle-based viral vaccine in eliciting cross-protective immune response in pigs, a potential large animal model.

## Introduction

Microencapsulation of vaccine agents and drugs in biodegradable polymer and its delivery in humans is an innovative approach to create more robust medications and vaccines in the 21^st^ century. The biocompatible and biodegradable polymer, PLGA [poly (lactide-co-glycolides)], is the US FDA approved material in the development of nanoparticle-based controlled release delivery system [1]. PLGA slowly degrades and releases the vaccine over a long-period of time, thus avoiding the need of a booster dose [2]. Being particulate in nature, the nanoparticle-mediated delivery promotes uptake of Ags by professional antigen presenting cells (APCs). Nanoparticle as a delivery vehicle to vaccines and drugs has been extensively evaluated in mouse models. However, there are limitations in the translation of novel rodent findings to improve human and food animal health [3]. Therefore, pig may serve as a useful large animal model for such research. Moreover, due to their physiological, anatomical, and immunological similarity to humans, pigs are already in use as an animal model system to study a few viral diseases [4]. Although, the current study is focused on a respiratory pathogen which infects pigs, and the vaccine evaluation strategy may help to consider pig as a model system.

Among the swine diseases, porcine reproductive and respiratory syndrome (PRRS) is highly devastating, causing an estimated economic loss of $664 million annually in the US [5]. This translates into $1.8 million losses per day annually. PRRSV infects pigs of all ages and is caused by a highly mutating, positive sense, single stranded RNA virus belongs to the family *Arteriviridae* [6]. PRRS in growing pigs causes anorexia, fever, respiratory distress, and enhanced susceptibility to secondary microbial infections; while in pregnant sows it is characterized by reproductive dysfunction and abortions [7]. Primary PRRSV permissive cells are alveolar macrophages (Mφs) [8]. PRRSV rapidly modulates the host innate immune response, such as dampens the NK cell cytotoxicity and reduces the IFN-α production, and upregulates immunosuppressive mediators from as early as two days post-infection [9]; which lead to poor adaptive immune response and delayed/weak virus neutralizing antibody response, resulting in PRRSV persistence. Nevertheless, due to high degree of constant genetic and antigenic variations, control of PRRS remains a challenge to the swine industry worldwide.

Optimal mucosal immunization induces protective immune response at both mucosal and systemic sites compared to systemic immunization [10]. Generation of protective IgA response is essential to reduce and/or prevent the entry of pathogens whose principle port of entry is through mucosal sites [11]. Mucosal immunization induces effective immune response at both local and distant effector sites. Particulate antigen delivery system facilitates the passage of Ags through mucosal barrier and leads to stimulation of the underlying mucosal immune cells [12]. Biodegradable microspheres made of Chitosan, PLGA, and liposome have been in use to deliver candidate vaccines to mucosal sites [13]. A study using nanoparticle-entrapped killed influenza virus vaccine administered with an adjuvant intranasally to mice, rabbits, and pigs elicited protective immune response, with better immunity induced in pigs by intranasal compared to intramuscular route of vaccination [14]. A single intranasal delivery of PLGA nanoparticle-entrapped *Schistosoma mansoni* Ags to mice elicited protective neutralizing antibody response in the lungs and blood [15].

Since late 1990 s, modified live PRRSV (PRRS-MLV) and killed virus vaccines are in use to control PRRS, but neither of them protects pigs completely against heterologous field viruses [16]. Like the field virus, PRRS-MLV also induces immunosuppression [17], [18]. Moreover, there are reports of reversion of vaccine virus into virulence leading to severe disease outbreaks [19], [20]. Although available killed PRRSV vaccines are safe, they are poorly immunogenic [21]. Thus, to control PRRS outbreaks innovative vaccine strategies are required. In the current study, killed PRRSV Ags were encapsulated in PLGA nanoparticles and characterized the candidate vaccine *in vitro* and in a pre- and -post-challenge study in pigs. Various immune correlates of protection were analyzed both at mucosal and systemic sites to show the evidences of cross-protective immunity.

## Materials and Methods

### Preparation of vaccine antigens and PLGA nanoparticle-killed PRRSV vaccine (Nano-KAg)

MARC-145 cell-monolayer was infected with the PRRSV VR2332 strain [22] at 0.001 MOI (multiplicity of infection), and the harvested infected cell culture fluid was clarified and subjected to ultracentrifugation (20% sucrose overlay) at 100,000×g for 2 hr at 4°C. The semi-purified viral pellet was suspended in PBS; titrated, inactivated by UV irradiation (at 254 nm UV-irradiation [EL series UV lamps, UVP, LLC (CA); 8 watt/115V – 60 Hz/0.32 Amps] for 1 hr), sonicated, and the protein content was estimated using the BCA kit (Biorad, CA). Viral inactivation was confirmed by cell culture immunofluorescence assay in MARC-145 cells. The viral pellet was aliquoted and stored at −70°C. Control antigen was prepared similarly using uninfected MARC-145 cells. Sterile precautions were followed throughout the antigen preparation and processing procedures to avoid any bacterial contamination.

Nanoparticles were prepared using standard double emulsion solvent evaporation technique [23], [24]. Briefly, 15% of PLGA 50/50 (750 mg) was dissolved in 5 mg of killed VR2332 proteins, homogenized at 6000 rpm for 90 seconds, then added to aqueous solution of 10% polyvinyl alcohol and homogenized. Finally, the preparation was stirred overnight and the washed nanoparticles were freeze-dried and stored at 4°C. The amount of entrapped PRRSV protein in the nanoparticles was determined as described previously [25]. The size and shape of nanoparticles was determined by scanning electron microscopy (Hitachi S-3500N).

### Characterization of Nano-KAg by confocal microscopy

Bronchoalveolar lavage fluid (BAL) collected from three 4–6 weeks old healthy SPF pigs was processed to isolate mononuclear cells (BAL-MNC) [26]. BAL-MNC (1×10^6^ cells per ml) were plated in a 24 well plate containing poly-L-lysine coated cover slips for 1 hr. Non-adherent cells were aspirated and the adherent cells were treated with freeze-dried Nano-KAg containing different concentrations of PRRSV proteins suspended in DMEM containing 10% FBS. Cells uninfected or infected with PRRSV (VR2332 strain) at 0.1 MOI for 12 hr was included as control. Cells were fixed in 3% paraformaldehyde for 15 min, permeabilized (0.1% Triton X-100) and blocked (PBS containing 5% BSA and 0.2% triton X-100) for 1 hr at room temperature (RT). Subsequently, treated with anti-PRRSV nucleocapsid specific mAb SDOW17 (Rural Technologies, Inc.,) and early endosome specific goat polyclonal anti-early endosome antigen 1 (EEA-1) IgG (Santa Cruz Biotechnology, Santa Cruz, CA), diluted in the dilution buffer (PBS containing 1% BSA and 0.1% triton X-100) for 1 hr at RT. Followed by treatment with goat anti-mouse IgG Alexa Flour488 and donkey anti-goat Alexa flour 633 (Invitrogen), and incubated for 1 hr at RT. Cells were washed in between the treatment steps and treated with a mounting medium containing 2.5% DABCO (Sigma). Stained coverslips were mounted on a clean glass slide using transparent nail polish and viewed under a Leica confocal microscope. The acquired images were analyzed using Leica confocal software.

### *In vitro* uptake of Nano-KAg by pig Mφs and determination of CD80/86 expression

BAL-MNC (1×10^6^ cells per ml) were seeded in a 24-well plate and untreated or treated with K-Ag or Nano-KAg (2, 0.2, and 0.02 µg/ml of PRRSV protein) and incubated for 3 hr at 37°C. Cells uninfected or infected with PRRSV (MN184 strain) at 0.1 MOI for 12 hr was served as control. Cells were treated with anti-PRRSV N' mAb followed by goat anti-mouse IgG Alexa Flour488, washed, and fixed before analysis. To assess the expression of CD80/86 on professional antigen presenting cells (APCs), BAL-MNC were treated as above for 16 hr at 37°C, washed and stained using biotinylated human CTLA4-mouse immunoglobulin fusion protein (Ancell, MN) and PE-conjugated CD172 (Southern Biotech) [27], followed by streptavidin percpCy5.5. Cells were fixed and analyzed using FACS Aria II (BD Biosciences) flow cytometer.

### Pigs and inoculations

Conventional large White–Duroc crossbred weaned specific-pathogen-free (SPF) pigs from three different litters at 3–4 wks of age were confirmed seronegative for PRRSV, porcine respiratory corona virus, transmissible gastroenteritis virus, and porcine Circo virus 2 antibodies. In a pre-challenge study, pigs (n = 9) were grouped randomly into three groups (n = 3 per group). Group I – unvaccinated (mock) pigs inoculated with DMEM and PBS; Group II – inoculated with K-Ag; Group III – inoculated with Nano-KAg. Each vaccine (Nano-KAg and K-Ag) dose has one mg of crude viral preparation containing ∼5×10^6^ TCID_50_ of inactivated virus. The vaccine was inoculated once, intranasally. All the pigs were euthanized on post-immunization day (PID) 15 and evaluated for innate and virus specific adaptive immune responses.

In a post-challenge study, pigs (n = 12) were divided randomly into four groups (n = 3 per group). Group I – mock pigs; Group II – inoculated with normal saline; Group III – inoculated with K-Ag; Group IV – inoculated with Nano-KAg. Each vaccine dose had same amount of Ags as described above. Groups II, III, and IV were challenged with PRRSV MN184 (0.5×10^6^ TCID_50_/ml_,_ 2 ml per pig) on PID 21 and euthanized on day post-challenge (DPC or PC) 15. All the inoculations were performed once by intranasal route. The dose of Nano-KAg (1 mg per pig) was chosen based on the results of a dose-dependent response study performed earlier in pigs. Mock-inoculated pigs were euthanized separately before sacrificing virus challenged animals. Pigs received food and water *ad libitum* and maintained under the supervision of a veterinarian. All the pigs were maintained, samples collected, and euthanized as per the standard procedures with necessary efforts to minimize suffering of animals. The animal use protocol was approved by the Committee on the Ethics of Animal Experiments of The Ohio State University.

### Ethics statement

This study was carried out in strict accordance with the recommendations by Public Health Service Policy, United States Department of Agriculture Regulations, the National Research Council's Guide for the Care and Use of Laboratory Animals, and the Federation of Animal Science Societies' Guide for the Care and Use of Agricultural Animals in Agricultural Research and Teaching, and all relevant institutional, state, and federal regulations and policies regarding animal care and use at The Ohio State University. The protocol was approved by the Committee on the Ethics of Animal Experiments of The Ohio State University (Protocol Number: 08-AG028). All the pigs were maintained, samples collected, and euthanized, and all efforts were made to minimize suffering of animals.

### Gross and histological analysis

During necropsy the lungs and lymph nodes were examined grossly and histologically. Macroscopic pulmonary lesions were given an estimated score based on the percentage of consolidated lesions in individual lobes as described previously [28]. The lung tissue samples collected from the caudal lobe was fixed in 10% neutral buffered formalin and sections (3 µm) made were stained for hematoxylin-and-eosin (H&E) as described previously [28]. Frozen lung sections were immunostained as described previously [29]. Briefly, sections were treated with PRRSV nucleocapsid protein specific mAb (SDOW17) or isotype control mAb followed by ABC peroxidase staining kit (Vectastain Elite, Vector Labs) and the labeling was visualized by application of DAB (3, 3′-diaminobenzidine) substrate (Vector Laboratories) and counterstained with hematoxylin. Immunostained slides were examined by an unbiased certified veterinary pathologist to score the presence of PRRSV Ags.

### Virus titration and Virus neutralizing test (VNT)

PRRSV titer and virus neutralizing antibody titer in serum and in the lung homogenate was analyzed by indirect immunofluorescence assay (IFA) as previously described [30].

### PRRSV specific isotype antibody analysis in the lungs and blood

PRRSV specific IgA and IgG antibodies in serum and lung lysate (homogenate) were analyzed by ELISA. Briefly, ELISA plates were coated with pre-titrated semi-purified killed PRRSV (MN184) Ags (10 µg/ml) in carbonate- bicarbonate buffer (pH 9.6), washed and blocked (1% BSA+0.1% Tween 20 in PBS). Serum (1∶100) and lung lysate (0.5 mg/ml, w/v) samples were added and incubated for 2 hr at RT. The bound virus specific isotype antibody was detected using anti-pig IgA and IgG secondary antibodies conjugated with HRP (KPL). Plates were developed using the chromogen TMB and read at 450 nm. We also included non-PRRSV antigen-coated plates as control and the OD values obtained from experimental plate were subtracted from the control.

### PRRSV specific cytokine response on antigen restimulation

Five million pig PBMC, TBLN (tracheobronchial lymph nodes) MNC, and lung MNC were subjected to *ex vivo* restimulation in the absence or presence of PRRSV MN184 Ags (50 μg/ml) as described previously [31], and the harvested supernatant was analyzed to measure cytokines. Cytokines secreted by immune cells cultured in the absence of PRRSV Ags was subtracted from the corresponding test value.

### Analysis of cytokine response and flow cytometric analysis of immune cells

Serum samples, harvested culture supernatants, and lung lysates were analyzed for Th1 (IFN-γ and IL-12), Th2 (IL-4), pro-inflammatory (IL-6), and immunosuppressive (IL-10 and TGF-β) cytokines by ELISA [31]. Amount of cytokines present in the lung lysate was normalized to picogram per gram of lung tissue. Flow cytometry analysis was performed to determine the phenotype and the frequency of different immune cells by a multicolor immunoassay as described previously [31]. Since MNC were isolated from different amounts (weights) of tissues (lungs and TBLN) we did not assess the absolute cell numbers. In this study, we determined relative frequency of individual immune cell subset by immunostaining fixed number of MNC (one million) from each site of collection, and 50,000 events were acquired in BD FACS Aria II (BD Biosciences) and analyzed using FlowJo software.

### Statistical analysis

All data were expressed as the mean of three pigs +/− SEM. Statistical analyses were performed using one way analysis of variance (ANOVA) followed by post-hoc Tukey's test using GraphPad InStat (software version 5.0) to establish differences among unvaccinated, K-Ag and Nano-KAg pig groups in post-challenge trial and between K-Ag and Nano-KAg pig groups in pre-challenge trial. Statistical significance was assessed as *P*<0.05.

## Results

### *In vitro* characterization of PRRSV entrapped-nanoparticles

**Figure 1:**
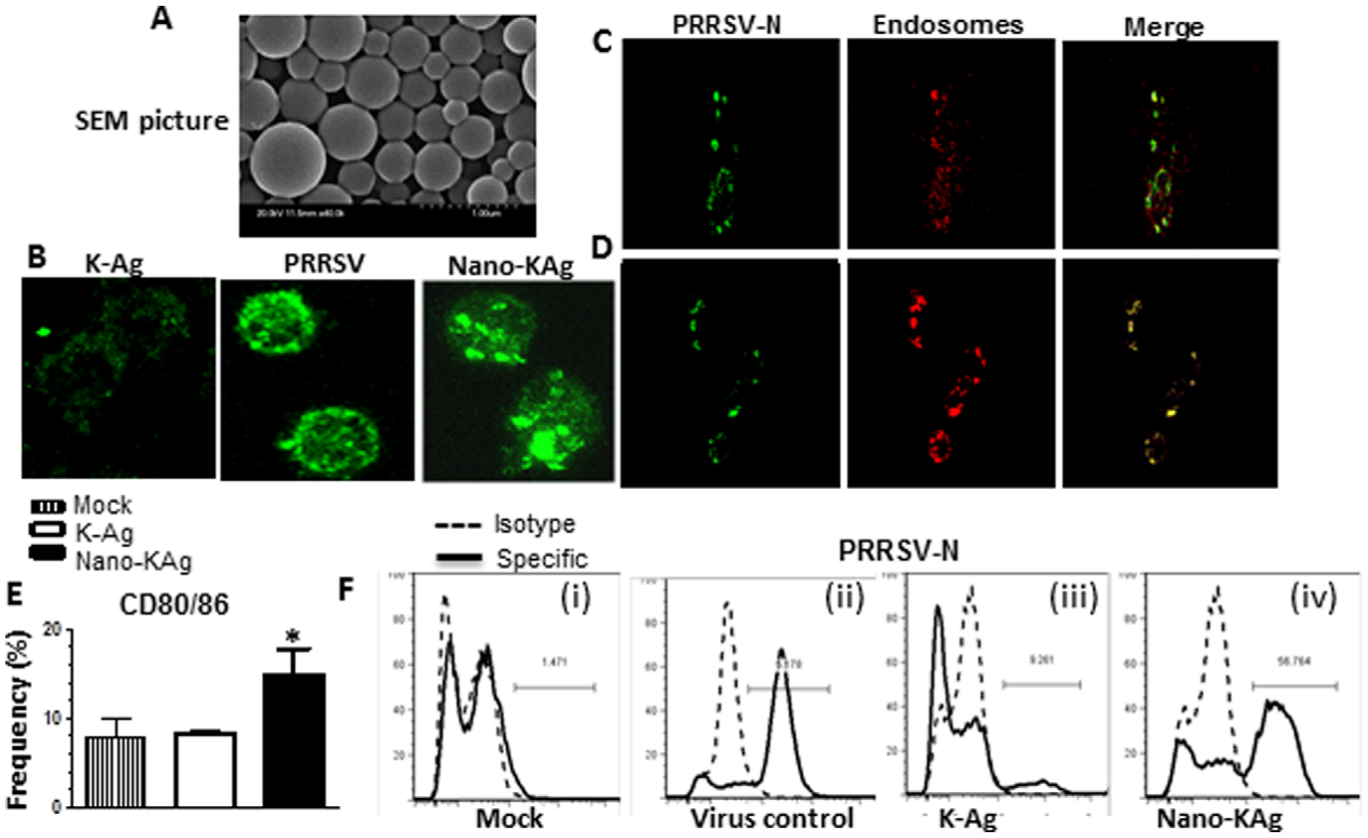
Characterization of Nano-KAg in pig alveolar Mφs. (A) Scanning electron micrograph of candidate Nano-KAg vaccine. Confocal microscopy pictures: (B) alveolar Mφs were treated with K-Ag, Nano-KAg, or infected with PRRSV and treated with PRRSV N' protein mAb and Alexa 488 anti-mouse IgG; co-localization of PRRSV N protein in endosomes of Mφs treated with Nano-KAg (C) and PRRSV infected (D). (E) Upregulation of CD80/86 expression on myeloid cells (CD172^+^) treated with Nano-KAg. Each bar represents average percent Mφs positive for CD80/86 in mock, K-Ag, and Nano-KAg treated BAL-MNC of three pigs +/− SEM. Asterisk represents the statistical significant difference (p<0.05) between Nano-KAg and K-Ag received pig groups. (F) Representative histogram showing the presence of PRRSV-N protein in Mφs, treated as indicated. Similar results were obtained in three independent trials.

Morphology of sham and PRRSV Ags entrapped PLGA nanoparticles was determined by scanning electron microscopy which revealed the size of particles as 200–600 nm (Figure 1, *A*). The average protein content in nanoparticles or core-loading was 0.50–0.55% (w/w), which represents an encapsulation efficiency of 50–55%. Upon re-dispersion of the Nano-KAg in PBS, PRRSV proteins were released slowly in the first 48 hr, later a gradual release profile was observed over the next 5-weeks (data not shown).

Uptake of Nano-KAg by APCs was studied using BAL-MNC harvested from three healthy SPF pigs. The confocal images of alveolar Mφs revealed preferential uptake of Nano-KAg but not unentrapped viral Ags (K-Ag), PRRSV infected cells served as a positive control (Figure 1, *B*). Engulfed nanoparticles delivered the PRRSV Ags to early endosomes and it was comparable to virus-infected control (Figure 1, *C & D*). Further, Nano-KAg engulfed APCs underwent maturation as indicated by significantly increased expression of CD80/86 (Figure 1, *E*). In addition, 57% of BAL-MNC treated with Nano-KAg was positive for PRRSV protein comparable to virus infected cells (Figure 1, *F ii & iv*). In contrast, only 9% BAL-MNC treated with K-Ag were positive for viral protein (Figure 1, *F iii*). Our results suggested that PRRSV Ags delivered in nanoparticles were phagocytosed by APCs and the released protein was found in the endosomes.

### Potential of PRRSV Ags entrapped nanoparticle (Nano-KAg) as a candidate vaccine

**Figure 2:**
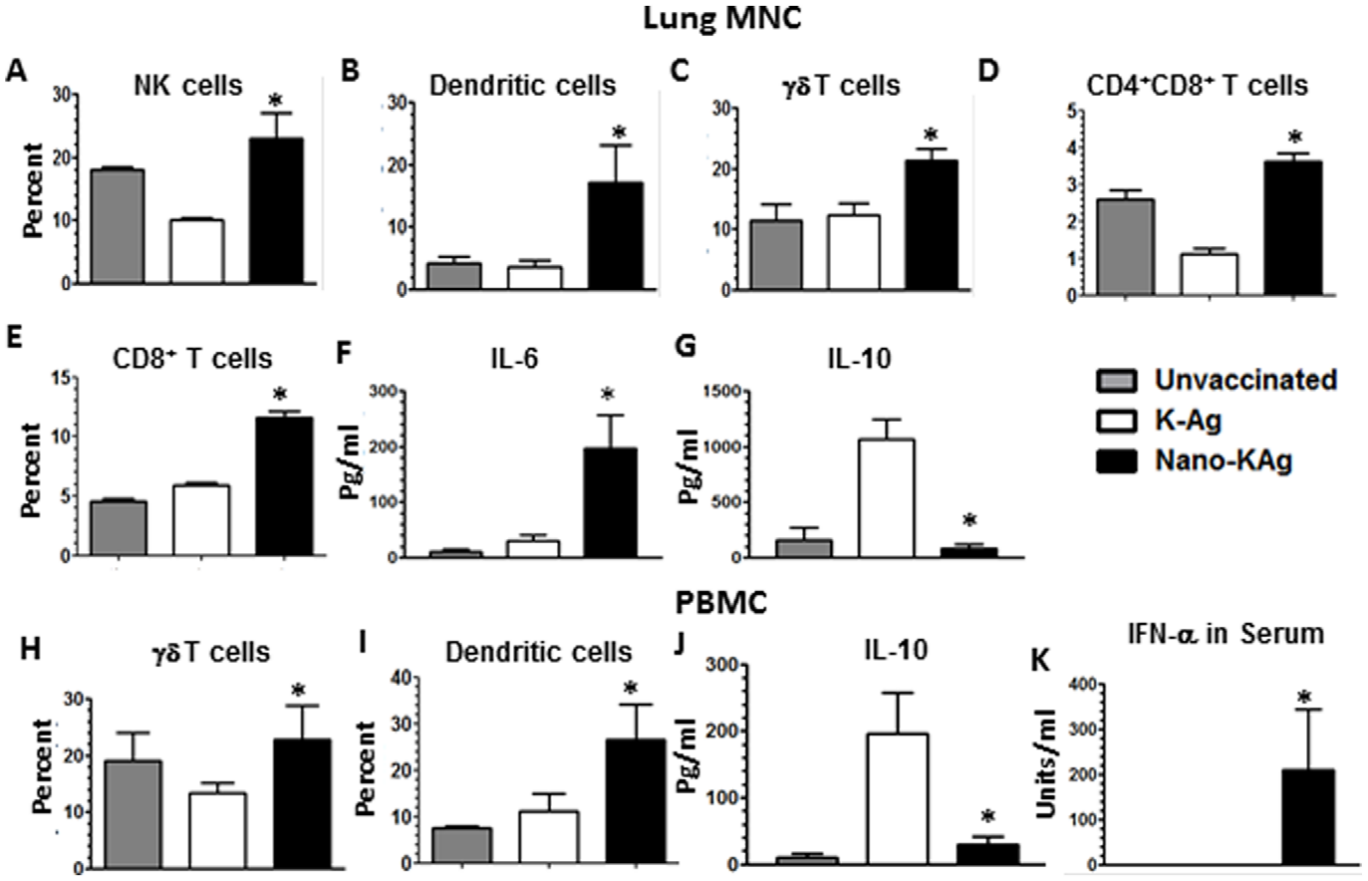
Nano-KAg elicited enhanced innate and suppressed regulatory response in a pre-challenge study. Pigs were unvaccinated or vaccinated as indicated. MNC were immunostained to analyze the frequency of immune cells: (A) NK cells, (B) Dendritic cells, (C) γδ T cells, (D) Th/memory cells, (E) CD8^+^ T cells in lung MNC; and (H) γδ T cells and (I) Dendritic cells in PBMC. Harvested culture supernatants from restimulated MNC were analyzed for cytokines: (F) IL-6 and (G) IL-10 in lung MNC; (J) IL-10 in PBMC; (K) IFN-α in serum by ELISA. Each bar represents the average cytokine amounts from three pigs ± SEM. Asterisk represents the statistical significant difference (p<0.05) between Nano-KAg and K-Ag received pig groups.

In a pre-challenge study, intranasal delivery of Nano-KAg resulted in induction of innate immune response at both mucosal and systemic sites, indicated by a significant increase in the frequency of NK cells, DCs, and γδ T cells in the lung MNC (Figure 2, *A–C*); and γδ T cells and DCs in the PBMC compared to K-Ag vaccinated pigs (Figure 2, *H & I*). Immune cells involved in adaptive arm of the immune response, such as CD4^+^ CD8^+^ T cells (Th/memory) and CD8^+^ T cells were increased significantly in the lung MNC of Nano-KAg compared to K-Ag vaccinated pigs (Figure 2, D *& E*). Further, lung MNC and PBMC from Nano-KAg immunized pigs secreted significantly reduced levels of the cytokine, IL-10, and higher amounts of IL-6 in a recall response (Figure 2, *F, G, & J*). In addition, innate cytokine IFN-α was secreted at significantly higher levels in pigs vaccinated with Nano-KAg compared to both the control groups (Figure 2, *K*).

### Significant reduction in the lung pathology and virus load in Nano-KAg vaccinated pigs

**Figure 3:**
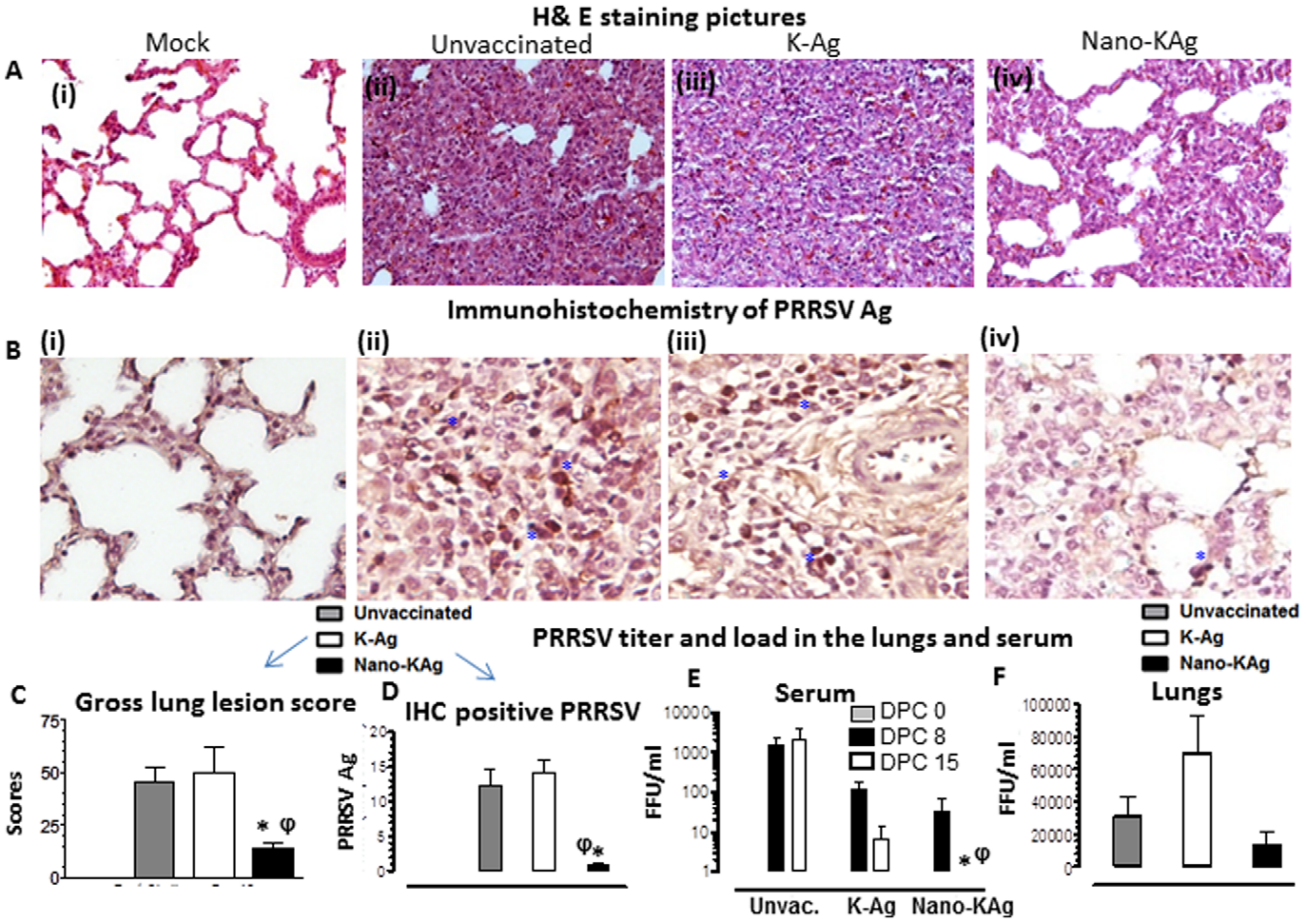
Reduced lung pathology and viral load in Nano-KAg vaccinated MN184 challenged pigs. Pigs were unvaccinated or vaccinated as indicated and challenged with PRRSV MN184 and euthanized at DPC 15. (A) A representative pig lung H&E picture from indicted pig group. (B) A representative lung immunohistochemistry (IHC) picture from indicted pig group showing PRRSV N antigen positive cells (asterisk). (C) Gross lung lesions were graded based on percent lung area affected and severity of inflammatory pathology. (D) PRRSV N antigen positive cells in IHC were counted in 10 random fields from each pig. The PRRSV titer in fluorescence foci units at indicated DPC in (E) serum and (F) in the lungs was determined by immunofluorescence assay. Each bar represents average values from three pigs ± SEM. Asterisk represents the statistical significant difference (p<0.05) between K-Ag and Nano-KAg received pig groups, and φ represents the statistical significant difference (p<0.05) between unvaccinated and Nano-KAg received pig groups. A similar trend in results was obtained in an independent second trial performed using same number of animals.

In a post-challenge study, Nano-KAg vaccinated MN184 challenged pigs were clinically healthy with no fever or respiratory distress. In contrast, both K-Ag and unvaccinated, MN184 challenged pigs had irregular fever with reduced feed intake during the first two-week post-challenge. Microscopic examination of H&E stained lung sections of unvaccinated and K-Ag vaccinated, MN184 challenged pigs' revealed severe pneumonic lesions with massive infiltration of mononuclear cells with large consolidated area. In contrast, significantly reduced lung lesions were observed in Nano-KAg vaccinated virus challenged pigs (Figure 3, *A*). Significantly reduced gross lung lesion scores in Nano-KAg immunized group compared to other two virus challenged groups was observed (Figure 3, *C*).

Immunohistochemistry analysis had revealed abundant PRRSV antigen positive cells in the lung sections of unvaccinated and K-Ag vaccinated, MN184 challenged pigs compared to Nano-KAg received pigs (Figure 3, *B & D*). PRRSV titer in serum samples indicated a reduced viral load of greater than one-log at PC 7 with complete viral clearance by PC 15 in Nano-KAg vaccinated, compared to K-Ag vaccinated and unvaccinated pigs (Figure 3, *E*). Similarly, PRRSV load in the lungs was also reduced (although not significant) in Nano-KAg compared to K-Ag immunized, MN184 virus challenged pigs (Figure 3, *F*). Also the PRRSV titer (TCID_50_/ml) in both the serum and lung homogenate showed a reduction in Nano-KAg immunized compared to control pigs (data not shown).

### Humoral immune response in serum, lungs and nasal swab

**Figure 4:**
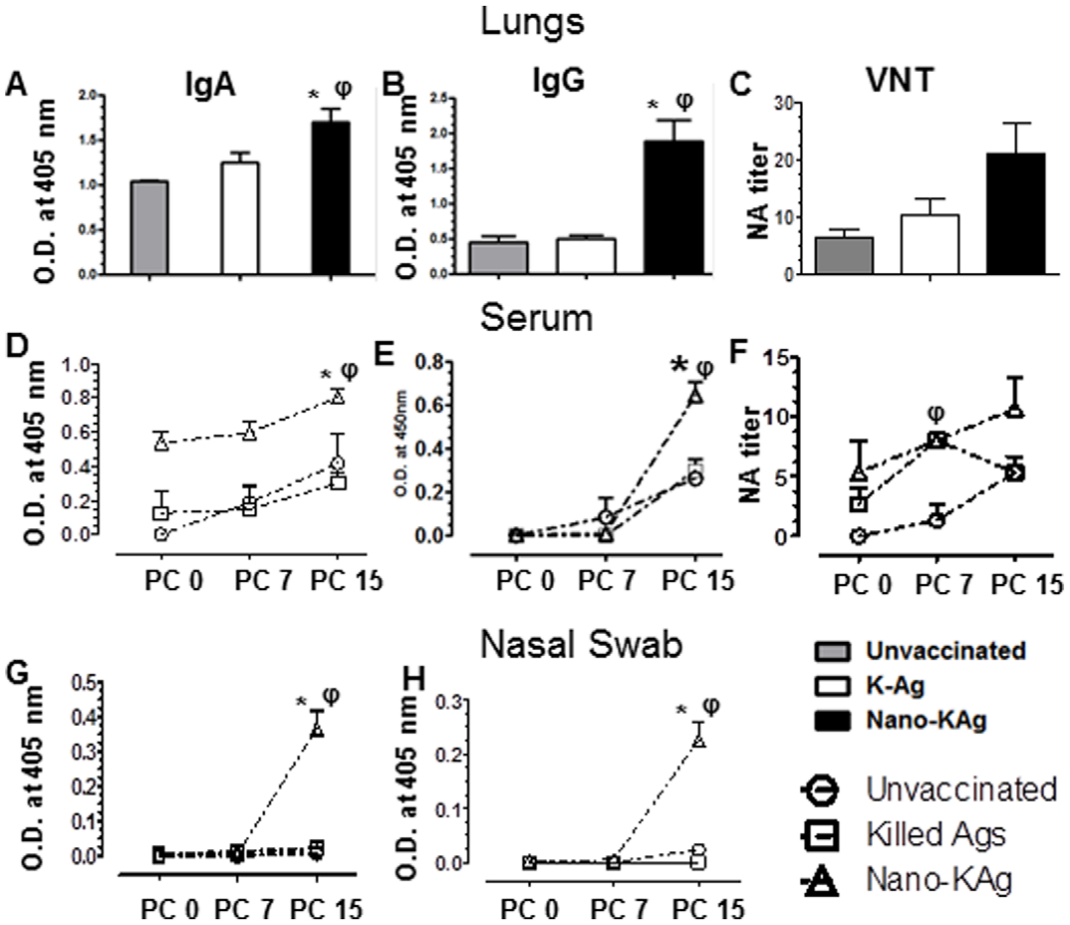
Enhanced PRRSV specific IgA and neutralizing antibody response in Nano-KAg vaccinated virus challenged pigs. Pigs were unvaccinated or vaccinated, challenged and euthanized as indicated in Fig 3 legend. Anti-PRRSV IgA and IgG antibody response in the (A&B) lungs, (D&E) serum, and (G&H) nasal swabs, was determined by ELISA. PRRSV neutralizing antibody response in (C) lungs and (F) serum was determined by immunofluorescence assay. Each bar represents average optical density value or VN titer from three pigs ± SEM. Asterisk represents the statistical significant difference (p<0.05) between K-Ag and Nano-KAg received pig groups, and φ represents the statistical significant difference (p<0.05) between unvaccinated and Nano-KAg received pig groups. A similar trend in results was obtained in an independent second trial performed using same number of animals.

Lung homogenates of Nano-KAg immunized pigs contained significantly higher levels of virus specific IgA and IgG antibodies compared to unvaccinated and K-Ag vaccinated, MN184 challenged pigs (Figure 4, *A & B*). In the serum samples of Nano-KAg vaccinated pigs increased IgA antibody levels at PC 0 (samples collected on the same day as viral challenge but before inoculating the challenge virus) with a significant increase at PC 15 compared to either unvaccinated or K-Ag vaccinated, MN184 challenged pigs was detected (Figure 4, *D*). The PRRSV specific IgG antibody levels in serum (Figure 4, *E*) and both IgA and IgG levels in the nasal swab (Figure 4, *G & H*) were significantly higher in Nano-KAg vaccinated, compared to unvaccinated and K-Ag immunized pigs at DPC 15. Significantly increased PRRSV specific neutralizing antibody (VN) titers in serum of both K-Ag and Nano-KAg vaccine received pig groups was observed at PC 7, which still remained high (although not significant) only in the Nano-KAg group at PC 15. In the lungs, a similar trend of increased (but not significant) titers of neutralizing antibodies in Nano-KAg immunized pigs was seen (Figure 4, *C & F*).

### Nanoparticle-based PRRSV vaccine showed enhanced innate immune response in pigs

**Figure 5:**
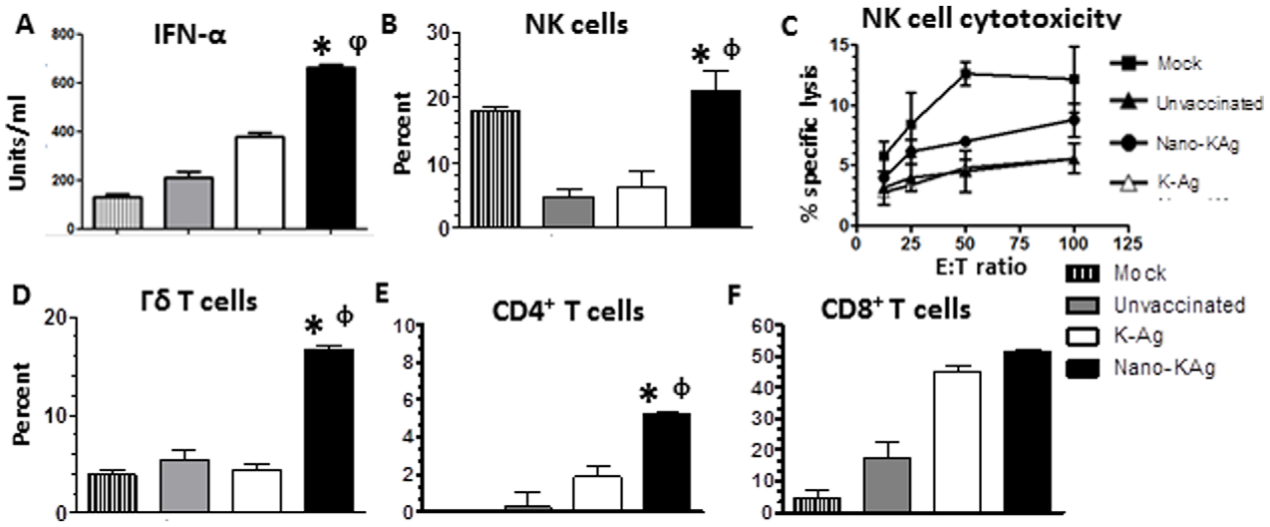
Nano-KAg elicited enhanced innate immune response in the lungs of pigs. Pigs were unvaccinated or vaccinated, challenged and euthanized as indicated in Fig 3 legend. (A) Lung homogenates were analyzed for the cytokine IFN-α by ELISA. Lung MNC were analyzed to determine the frequency of (B) NK cells, (D) γδ T cells, (E) CD4^+^ T cells, and (F) CD8^+^ T cells by flow cytometry. (C) Lung NK cells were analyzed for cytotoxicity by LDH assay. Each bar or data point in the graph represents average values from three pigs ± SEM. Asterisk represents the statistical significant difference (p<0.05) between K-Ag and Nano-KAg received pig groups, and φ represents the statistical significant difference (p<0.05) between unvaccinated and Nano-KAg received pig groups. A similar trend in results was obtained in an independent second trial performed using same number of animals.

Nano-KAg vaccine received MN184 virus challenged pigs had significantly increased innate IFN-α production in the lungs (Figure 5, *A*). In K-Ag vaccinated and unvaccinated pigs, a four-fold reduction in NK cell frequency compared to mock pigs was observed. In contrast, in Nano-KAg vaccinated pigs the NK cell frequency was significantly higher than the K-Ag and unvaccinated virus challenged pigs (Figure 5, *B*). Further, lung NK cell-cytotoxic function in unvaccinated and K-Ag vaccinated, MN184 virus challenged pigs was completely suppressed; however, in Nano-KAg received pigs it was partially rescued (Figure 5, *C*). The frequency of γδ T cells and CD4^+^ (but not CD8^+^) T cells in the lungs of Nano-KAg vaccinated animals were significantly increased compared to K-Ag and unvaccinated, virus challenged pigs (Figure 5, *D, E & F*). In the peripheral blood of Nano-KAg immunized pigs a significantly increased frequency of DCs, and in TBLN significantly increased frequency of both DCs and γδ T cells was observed (Table 1).

**Table 1:** Frequency of immune cells in PBMC and TBLN of Nano-KAg vaccinated pigs.

Immune cells	Mock	Unvaccinated	K-Ag	Nano-KAg
**PBMC**				
Myeloid cells	21.1±0.5	59.3±4.3	69.9±3.7	72.8±2.5
Dendritic Cells^ a^	3.1±0.6	0.01±0.3	0.7±0.1	3.03±0.6*
γδ T cells	2.2±0.5	1.1±0.1	2.7±0.3	4.7±0.7
NK cells^ b^	4.1±0.3	32.6±10.0	13.2±1.6	16.4±1.6
Tregs ^c^	0.3±0.1	0.9±0.2	1.8±0.1*	0.9±0.1
**TBLN MNC**				
Myeloid cells	1.7±0.8	18.2±0.6	15.9±3.6	18.6±5.2
Dendritic Cells	1.5±0.0	0.8±0.1	0.1±0	5.6±1.2*
γδ T cells	1.8±0.1	1.1±0.4	1.5±0.3	8.6±0.9*
NK cells	5.1±0.6	3.7±0.3	1.3±0.2	8.5±3.7
Tregs	1.3±0.7	3.1±0.2	2.1±.02	1.8±0.04

Pigs were unvaccinated or vaccinated with either K-Ag or Nano-KAg once intranasally and challenged with PRRSV strain MN184 and euthanized at DPC 15. Different immune cell subsets present in PBMC and TBLN MNC were enumerated by flow cytometry. ^a^ CD172^+^ cells were gated to enumerate CD11c and SLAII expression and the percent of DCs rich fraction (CD172^+^CD11c^+^SLAII^+^) is shown.^ b^ CD3^−^ cells were gated to enumerate CD4 and CD8α expression and the percent NK cell rich fraction (CD3^−^CD4^−^ CD8α^+^) is shown. ^c^ CD25^+^ cells were gated to enumerate CD4 and Foxp3 expression and the percent of CD4^+^CD25^+^Foxp3^+^ cell is shown. Each number is an average percent of immune cells from three pigs +/− SEM. Asterisk represents the statistical significant difference (p<0.05) between Nano-KAg and K-Ag received pig groups.

### Suppression of immunosuppressive cytokine with boosting of IFN-γ response by Nano-KAg

**Figure 6:**
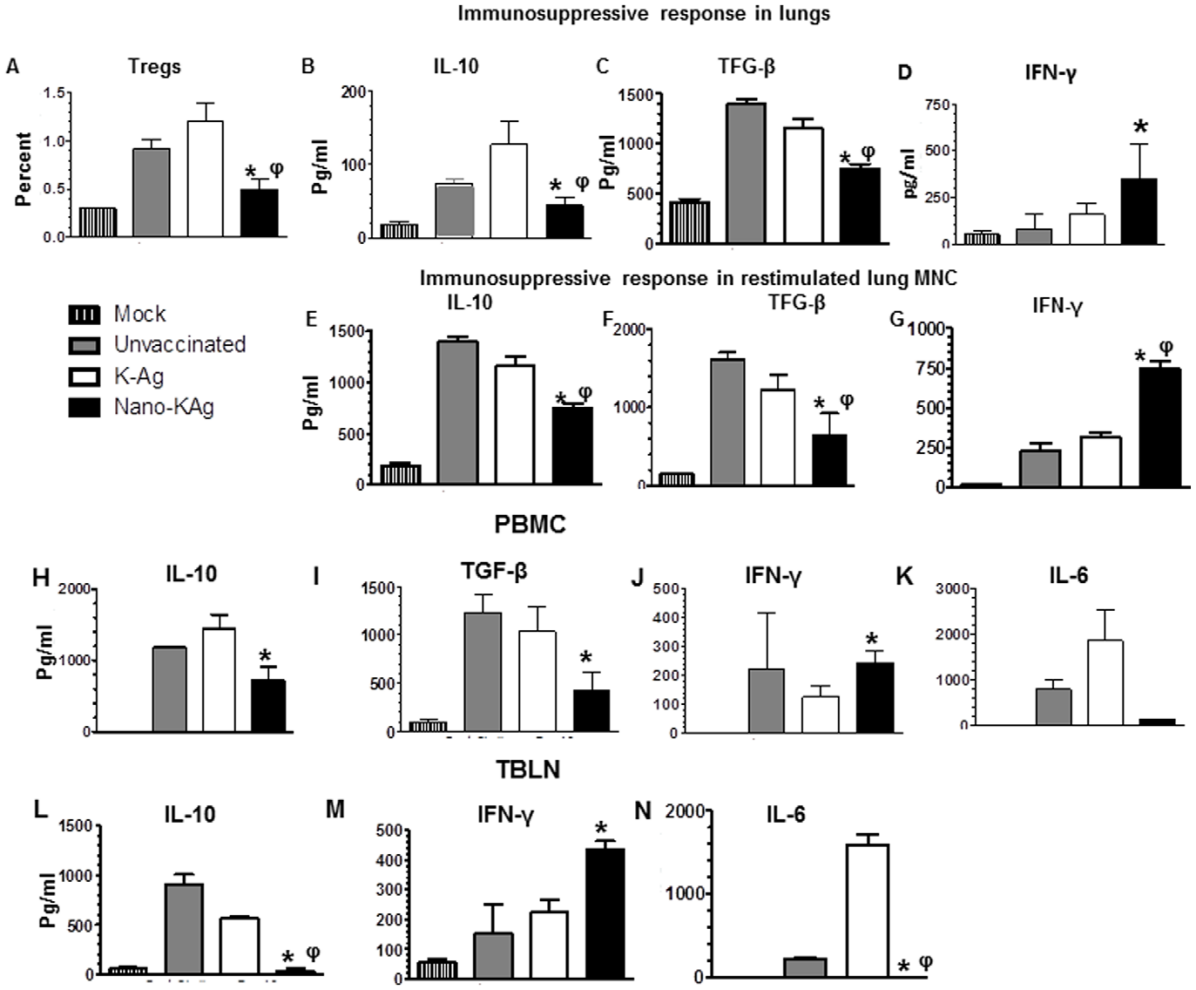
Reduction in the immunosuppressive and increased Th1 cytokines response in Nano-KAg vaccinated MN184 challenged pigs. Pigs were unvaccinated or vaccinated, challenged and euthanized as indicated in Fig 3 legend. (A) Lungs MNC were analyzed for Tregs population by flow cytometry. Lung homogenates were analyzed for cytokines: (B) IL-10, (C) TGF-β, and (D) IFN-γ; and harvested culture supernatants from restimulated lung MNC were analyzed for cytokines: (E) IL-10, (F) TGF-β, and (G) IFN-γ by ELISA. Similarly harvested culture supernatants from restimulated PBMC and TBLN MNC were analyzed for cytokines: (H & L) IL-10, (I) TGF-β, (J & M) IFN-γ, and (K & N) IL-6 by ELISA. Each bar represents average values from three pigs ± SEM. Asterisk represents the statistical significant difference (p<0.05) between Nano-KAg and K-Ag received pig groups and φ represents the statistical significant difference (p<0.05) between unvaccinated and Nano-KAg received pig groups. A similar trend in results was obtained in an independent second trial performed using same number of animals.

Pigs vaccinated with Nano-KAg had significantly reduced Foxp3^+^ T-regulatory cell (Treg) population in the lungs, compared to unvaccinated and K-Ag received pigs (Figure 6, *A*). Immunosuppressive cytokines (IL-10 and TGF-β) response in the lungs was significantly reduced in Nano-KAg vaccinated pigs; and also their decreased secretion was detected in lung MNC restimulated with killed MN184 Ags (Figure 6, *B, C, E & F*). In contrast, a significantly increased IFN-γ in the lung homogenate, and its secretion in antigen restimulated lung MNC was detected in Nano-KAg vaccinated pigs (Figure 6, *D & G*). In addition, PBMC secreted significantly reduced IL-10 and TGF-β compared to K-Ag vaccinated virus challenged pigs (Figure 6, *H & I*), while in TBLN-MNC, increased IL-10 was seen compared to both virus challenged groups (Figure 6, *L*). Increased IFN-γ was secreted in both PBMC and TBLN-MNC of Nano-KAg vaccine group compared to K-Ag immunized pigs (Figure 6, *J & M*). The level of proinflammatory cytokine, IL-6, in a restimulation response was significantly reduced in TBLN-MNC of Nano-KAg compared to both virus challenged pigs (Figure 6, K & N).

## Discussion

Nanoparticle mediated vaccine delivery has shown a great promise in mouse models against influenza, parainfluenza, hepatitis B, plasmodium, and venezuelan equine encephalitis pathogens [13], [32], [33]. However, the knowledge related to cross-protective efficacy of such vaccines in a suitable large animal model is limited. Our study has revealed the potency of nanoparticle-entrapped PRRSV vaccine in pigs.

PRRS has been a dreadful disease causing huge economic loss in a majority of the swine producing countries in the world. Nano-KAg vaccine has upregulated the frequency of major innate immune players (NK cells, γδ T cells, and DCs), and also enhanced the secretion of anti-viral cytokines (IFN-α and IFN-γ), which otherwise is suppressed by PRRSV [34]. Suggesting that in Nano-KAg vaccinated virulent heterologous PRRS challenged pigs, these effectors played a pivotal role in the viral clearance. In contrast, increased viral load in the lungs of K-Ag vaccinated pigs was perhaps due to antibody-mediated enhancement in the uptake of PRRSV by alveolar Mφs, in addition to increased immunosuppressive response.

Particulate Ags has an inherent affinity for mucosal M cells and APCs and are phagocytosed passively by APCs [35]. Nanoparticles protect the entrapped proteins from protease-mediated degradation at mucosal surfaces, and also aid in slow sustained release of the vaccine [36]. PLGA nanoparticle induces activation and maturation of human DCs by upregulating the expression of costimulatory and MHC class II molecules, secretion of proinflammatory cytokines, and enhances the APCs allostimulatory property [37], [38]. Consistent with the inherent adjuvant property of PLGA, pig APCs treated with PLGA nanoparticle vaccine (Nano-KAg) had increased expression of a costimulatory molecule, CD80/86. In our study, we did not include a pig group with empty PGLA nanoparticles, as our primary goal was to augment the killed PRRSV vaccine induced anti-PRRSV cross-protective immunity with the help of PLGA, a well-known adjuvant and a delivery system to vaccines. Thus, at present we do not have the information on how much of post-vaccination T cell immunity detected in Nano-KAg vaccinated pigs was due to the adjuvant effect of the PGLA nanoparticles alone, which will be considered during our future investigations. However, a rapid uptake of Nano-KAg by lung APCs followed by translocation of viral Ags into endosomal compartment was observed. Suggesting that virus specific adaptive immune response could be elicited in the respiratory tract of pigs using PLGA nanoparticle-based killed PRRSV vaccine. Differential cell counts from BAL fluid harvested from healthy mice, humans, and pigs have indicated that greater than 90% of cells are alveolar Mφs [39], [40], suggesting that intranasally delivered Nano-KAg were phagocytosed by Mφs.

Earlier studies have demonstrated rapid uptake of Chitosan nanoparticles by APCs followed by gradual release of antigen due to slow rate of degradation of Chitosan by lysozymes [41]; resulting in increased expression of costimulatory molecules, activation of DCs, and antigen presentation through MHC class I and II molecules [42]. In our pre-challenge study, Nano-KAg vaccine significantly increased the frequency of CD8^+^ T cells, Th/memory cells, with concomitant increase in the secretion of innate (IFN-α), proinflammatory (IL-6), and Th1 (IFN-γ) cytokines. Immune potentiating ability of Chitosan nanoparticles is mediated by the action of innate immune cells, in addition to enhanced production of IL-6 and IFN-γ [43]. Phagocytosis of polystyrene latex microspheres by Mφs activate the signal transduction events in innate immune cells [44]. Once activated, the APCs present the antigen through MHC class I and II molecules to CD8+ and CD4+ T cells, respectively.

In our post-challenge study using a virulent heterologous PRRSV, Nano-KAg induced superior innate immune response was observed. Studies have shown immune potentiating activity of nanoparticles in mice, pigs, and macaques; but the immune correlates were not evaluated in vaccinated virus challenged animals [45]. Immunologically, PRRSV modulates innate immune function of pigs by dampening the IFN-α production and NK cell frequency as well as its cytotoxicity, leading to weak/delayed adaptive immune response [17], [34]. The NK cytotoxic function in Nano-KAg received pigs was partially rescued (not significant), suggesting the beginning of appearance of NK cell cytotoxic function with a complete rescue in their frequency (comparable to mock pigs). In pigs vaccinated with Nano-KAg, virus induced immunosuppressive responses were dampened along with a significant boost in both innate and virus specific adaptive immune response.

The γδ T cell is an important innate immune cell at mucosal sites and they possess non-MHC class I cytolytic activity. Pigs possess relatively large population of γδ T cells compared to other species and they secrete IFN-γ [46]. γδ T cell plays an important role in reducing the vaccinia virus load, and also in destruction of herpes simplex type 1 infected cells [47]. In Nano-KAg vaccinated pigs increased population of γδ T cells, in addition to NK cells, CD4^+^ and CD8^+^ T cells, and increased secretion of IFN-γ were detected at both mucosal and systemic sites.

Now and earlier we have demonstrated that both K-Ag and MLV-PRRS vaccinated and virus challenged, as well as unvaccinated PRRSV infected pigs have immunosuppressed response mediated by increase in the population of Tregs and secretion of IL-10 and TGF-β, and reduced production of IFN-γ [17], [31], [48]. In contrast, in Nano-KAg immunized pig lungs, TBLN, and blood significantly reduced Tregs frequency, associated with decreased IL-10 and TGF-β, and increased IFN-γ secretion, compared to unvaccinated and K-Ag vaccinated virus challenged pigs was observed. In a pre-challenge study, at two-week post-vaccination increased secretion of proinflammatory cytokine, IL-6, in Nano-KAg vaccinated pigs appears to be involved in initiation of adaptive immune response. Diminished production of IL-6 in post-challenged pigs at six-week post-vaccination was associated with reduced inflammatory lung pathology.

Mucosal immunization elicits production of IgA antibodies and effector response at distant tissues [49]. The IgA antibody is protective against various viral infections and they possess significant virus neutralization activity at both mucosal surfaces and blood [50]. In Nano-KAg immunized pigs increased levels of PRRSV specific IgA, IgG in the lungs, blood, and nasal wash were observed. Although, these PRRSV specific antibodies did not significantly increase the neutralizing titers in the lungs, there was complete clearance of viremia at PC 15, which was associated with various other adaptive immune correlates. Whereas in the lungs, the reduced viral titer was not significant, but a significant reduction of PRRSV antigen by immunohistochemistry and reduced lung pathology implicates the presence of active anti-PRRSV mucosal immune response elicited by Nano-KAg vaccine in the lungs (Fig 3 & 4).

The most important finding from our study to swine farmers and researchers is that pigs vaccinated intranasally with Nano-KAg vaccine completely clear the PRRSV viremia of a virulent heterologous virus by two-week post-challenge. Further, in yet another Nano-KAg vaccine study we inoculated a booster dose of the vaccine and co-administered with a potent mucosal adjuvant, intranasally, showed the complete clearance of the replicating heterologous PRRSV from the lungs and clearance of viremia earlier than the current study (Binjawadagi et al., manuscript submitted). In conclusion, our study has suggested that innovative strategy of intranasal delivery of PRRSV Nano-KAg vaccine has the potential to control PRRS outbreaks, and it has the potential to reduce economic losses to swine producers. Considering pig a useful large animal model, our study may serve as a useful impetus to undertake intranasal PLGA nanoparticle-based vaccine trials against human respiratory viral infections.
